# Llegaron las vacunas: ¿estamos listos?

**DOI:** 10.7705/biomedica.6072

**Published:** 2021-03-19

**Authors:** Fernando Ruiz, Julián Fernández-Niño

**Affiliations:** Ministro de Salud y Protección Social

La vacunación de más de 35 millones de personas contra el coronavirus durante el 2021 es, sin duda, una de las principales pruebas del sistema de salud colombiano en toda su historia. Durante el 2020, este fue capaz de expandir sus unidades de cuidados intensivos, mantener su capacidad de respuesta y funcionar adecuadamente, a pesar de evidentes diferencias regionales que son la manifestación de las desigualdades históricas y de las deficiencias en aspectos claves como la realización de pruebas a las personas en la cadena de contactos. 

Los resultados en salud durante la pandemia en Colombia son consecuencia de los factores estructurales determinantes de la salud, la percepción del riesgo en el tiempo por parte de la población y el consecuente cumplimiento del autocuidado, así como de las medidas colectivas adoptadas por el gobierno nacional y las autoridades locales, las características sociodemográficas y las condiciones de salud de nuestra población. Solo en última instancia tales resultados dependen de la respuesta del sistema de atención en salud que, a pesar de ser la pieza final en el engranaje, ha hecho un gran aporte a la lucha contra el virus. Puede decirse que en el país la atención efectiva en salud se ha garantizado en la inmensa mayoría de los casos de Covid-19, incluidos los más graves. En ese sentido, debe anotarse que, aunque la letalidad es alta, el objetivo del sistema de atención en salud no se centra únicamente en salvar las vidas, sino también, en tratar a todas las personas con dignidad, esforzándose por ofrecer los servicios con equidad y calidad.

Entre los críticos razonables –y entre los que no lo son tanto– ha habido mucho escepticismo sobre las capacidades del país y del sistema de salud para lograr el objetivo de la vacunación contra la Covid-19. Todos somos conscientes de que conseguirlo marcará un hito por los muchos obstáculos logísticos, burocráticos y sociales que habrá que superar en un país complejo y diverso donde la geografía, las diferencias en las capacidades locales y la velocidad con la que debemos vacunar exigen desplegar una operación de planeación extremadamente compleja en todos los niveles, que requiere una rigurosa articulación de todos los involucrados, pues cada uno de ellos es determinante en el logro de las metas. Debemos señalar, sin embargo, que varios aspectos del Plan Nacional de Vacunación contra la Covid-19 permiten expresar un optimismo prudente. Entre ellos, se destacan los siguientes:

- *Marco jurídico sólido.* Además de haberse construido con base en la evidencia y el consenso científico, el Plan Nacional de Vacunación hace una declaración explícita de los principios que lo rigen y de la priorización que orienta las decisiones subsecuentes. En el centro de todo están la equidad, la solidaridad y la primacía del interés general. El Plan se encuadra en el marco constitucional y la Ley Estatutaria en Salud con base en el principio de igualdad material: tratar como iguales a los iguales, tratar como diferentes a los diferentes. Por consiguiente, la conjunción entre la bioética y la evidencia científica es el fundamento del Plan.

El Decreto 109 de 2021 refleja un cuidadoso esfuerzo técnico y, además, democrático. Se trata del único plan de vacunación contra la Covid de la región que contó con los aportes de los ciudadanos. El proyecto de decreto estuvo disponible cuatro días para comentarios, durante los cuales se recibieron más de 400 observaciones de organizaciones sociales, asociaciones científicas, agremiaciones médicas y de la ciudadanía en general. Todas las observaciones fueron respondidas y tenidas en cuenta, de manera que el plan que emergió es mucho mejor que la propuesta original, pero, además, es dinámico, está abierto a la actualización científica y es adaptable a contextos diferentes.

**-** *Priorización basada en criterios científicos.* Actualmente, las vacunas contra el coronavirus son bienes escasos de alto valor social. Su asignación no podría hacerse de forma arbitraria, no solamente porque sería ineficiente, sino porque podría profundizar las inequidades existentes, retrasar el impacto observable de la vacunación e, incluso, derivar en conflictos sociales.

El plan no define el orden de asignación de la vacuna, como algunos lo han malinterpretado, según el supuesto valor social de las personas, sino con base en objetivos epidemiológicos. Por ejemplo, siendo el objetivo de la fase 1 reducir la mortalidad e incidencia de los casos graves, se determinaron con base en la evidencia científica los grupos con mayor mortalidad y ello se tradujo en una selección que responde, en primer lugar, a la edad, la cual es una variable identificable sujeta a muchos menos errores de medición que el estado de salud, por lo que se definió la inclusión de los adultos mayores en las etapas 1 y 2. Razonamientos similares respaldaron la escogencia de otros grupos con gran exposición al virus, comenzando con el talento humano en salud y el personal de apoyo logístico indispensable para mantener la respuesta. Nadie está excluido del Plan, pero hay un orden para recibir la vacuna, el cual está determinado por el beneficio colectivo.

Es importante entender, por ejemplo, que la prioridad en la vacunación de los adultos mayores, aunque naturalmente los favorece a ellos, también redunda en mayores beneficios humanos –que son los más importantes–, sociales y económicos, pues disminuye la carga que recae sobre el sistema de salud y la necesidad de las restricciones de movilidad generalizadas que tanto impacto social tienen.

- *Capacidad adaptativa del plan y experiencia con el Plan Ampliado de Inmunizaciones.* Si bien los recursos del Plan Ampliado de Inmunizaciones no son suficientes para incorporar la vacuna contra el coronavirus, la experiencia en su implementación sí resulta muy valiosa. El Plan Ampliado de Inmunizaciones en Colombia ha llegado a todos los rincones del país y en su marco las coberturas de vacunación se han mantenido históricamente altas. Este Plan representa una fortaleza institucional que transciende los gobiernos, en él se reflejan el conocimiento y la experiencia de cientos de personas que han logrado coberturas efectivas contra más de 20 enfermedades inmunoprevenibles. Esa experiencia debe aprovecharse ahora, expandiendo, claro está, su capacidad.

El Plan Nacional de Vacunación contra la Covid-19 se adapta a los diferentes contextos, como las áreas rurales dispersas, y se puede actualizar conforme surja nueva evidencia científica. Un ejemplo de ello es, sin duda, la decisión de unificar etapas en el Amazonas ante la emergencia del linaje P1 en Brasil, lo que demuestra la capacidad de cambio rápido ante problemas nuevos. No se trató, en este caso, de priorizar una región, sino de proteger a todo el país.

- *Portafolio diversificado y distribución técnica.* Colombia optó por un portafolio de diversas vacunas que permiten su aplicación en contextos diferentes. Esto facilita avanzar en la cobertura en regiones donde la geografía dificultaría la aplicación de aquellas que requieren ultracongelación. Se trata de un esfuerzo para garantizar el flujo continuo de las vacunas y una distribución basada en criterios explícitos, totalmente desligada de juicios subjetivos o cálculos políticos.

- *Articulación de entidades territoriales y redes de prestación de servicios.* La pandemia incentivó la consolidación de redes, como las que se formaron para la asignación centralizada de las unidades de cuidados intensivos en varias ciudades. Esta capacidad de articulación y la confianza mutua han dado buenos resultados, lo que puede aprovecharse para que las redes de atención, bajo el liderazgo de los entes territoriales, se organicen mejor, con el fin de optimizar la vacunación, sobre todo en la fase 2, cuando esta deberá masificarse.

Podríamos hacer una lista similar de factores importantes frente a las amenazas y retos de este Plan Nacional de Vacunación, muchos de ellos ya reconocidos por las voces más críticas. Este proceso no será nada fácil. Somos plenamente conscientes de lo que enfrentamos y estamos listos para adaptar la operación al aprendizaje y a las necesidades que surjan. La planeación no puede ser fija, debe responder a la evolución de las necesidades. Debemos detectar estas amenazas para prevenirlas o darles respuesta efectiva en cada momento.

La implementación exitosa de este Plan Nacional de Vacunación contra el coronavirus depende de la armonización de todos los integrantes del sistema y de poner en el centro de nuestros esfuerzos a las personas, bajo el imperativo supremo de facilitar el acceso efectivo y oportuno de todas ellas en cada fase y etapa del Plan. Deben adelantarse acciones afirmativas para incluir a los que históricamente han sido excluidos y no dejar a nadie atrás. La pandemia nos ha impuesto a todos, personas e instituciones, la obligación de dar lo mejor de nosotros mismos. Hoy estamos en un punto crucial y no podemos fallar.

Dirección de Epidemiología y Demografía, Ministerio de Salud y Protección Social

